# Novel cellular senescence-related risk model identified as the prognostic biomarkers for lung squamous cell carcinoma

**DOI:** 10.3389/fonc.2022.997702

**Published:** 2022-11-17

**Authors:** Xiaoshan Hu, Liyi Guo, Guihong Liu, Zili Dai, Li Wang, Jian Zhang, Jun Wang

**Affiliations:** ^1^ Department of Internal Medicine of Oncology, Affiliated Cancer Hospital & Institute of Guangzhou Medical University, Guangzhou, China; ^2^ Department of Oncology and Hematology, The Sixth People’s Hospital of Huizhou City, Huiyang Hospital Affiliated to Southern Medical University, Huizhou, China; ^3^ Department of Radiation Oncology, Donguan Tungwah Hospital, Dongguan, China; ^4^ Department of Radiation Oncology, State Key Laboratory of Respiratory Diseases, Guangzhou Institute of Respiratory Disease, Affiliated Cancer Hospital & Institute of Guangzhou Medical University, Guangzhou, China; ^5^ Guangzhou Medical University, Guangzhou, China; ^6^ Department of Interventional Radiology, Affiliated Cancer Hospital & Institute of Guangzhou Medical University, Guangzhou, China

**Keywords:** senescence, immune microenvironment, immunotherapy, lung squamous cell carcinoma, overall survival

## Abstract

**Background:**

Lung cancer is one of the top causes of cancer-related death worldwide. Cellular senescence is a characteristic of cell cycle arrest that plays a role in carcinogenesis and immune microenvironment modulation. Despite this, the clinical and immune cell infiltration features of senescence in lung squamous cell carcinoma (LUSC) are unknown.

**Methods:**

The Cancer Genome Atlas (TCGA) and Gene Expression Omnibus (GEO) were used to get RNA-seq data and clinical information for LUSC. The least absolute shrinkage and selection operator (LASSO)-Cox regression, receiver operating characteristic (ROC), and Kaplan-Meier analysis were used to evaluate a risk model for predicting overall survival based on six differentially expressed genes. The tumor microenvironment (TME) and immunotherapy response were also studied.

**Results:**

To discriminate LUSC into high- and low-risk subgroups, a risk model comprised of six cellular senescence-related genes (CDKN1A, CEBPB, MDH1, SIX1, SNAI1, and SOX5) was developed. The model could stratify patients into high-risk and low-risk groups, according to ROC and Kaplan-Meier analysis. In the TCGA-LUSC and GSE73403 cohorts, the high-risk group had a worse prognosis (P<0.05), and was associated with immune cell inactivation and being insensitive to immunotherapy in IMvigor210.

**Conclusions:**

We discovered a new LUSC classification based on six cellular senescence-related genes, which will aid in identifying patients who will benefit from anti-PD-1 treatment. Targeting senescence-related genes appears to be another option for improving clinical therapy for LUSC.

## Introduction

Lung cancer ranks as one of the major causes of cancer-related mortalities globally, with an approximate 5-year survival rate of 16.6% (1, 2). Non-small-cell lung cancer (NSCLC) and small-cell lung cancer (SCLC) are the two most common types of lung cancer (3–5). One subtype of NSCLC is lung squamous cell carcinoma (LUSC), which accounts for about 40% of lung cancer cases (6–8). LUSC is associated with poor outcome and lacks accessible targeted therapies as compared to lung adenocarcinoma (9). As a result, finding possible biomarkers and elucidating their mechanisms in the development and progression of LUSC is critical.

Cellular senescence is a stress-induced process that results in irreversible cell cycle arrest. Recent studies have also identified that various processes, including oncogene activation (10–12), radiation (13, 14), chemotherapy (15, 16), and mitochondrial dysfunction (17, 18), were associated with the development and progression of cellular senescence. Apart from physiological roles for cellular senescence during tissue development, accumulating evidence have revealed that senescence was also related to pathological process, including atherosclerosis (19, 20), wound healing (21, 22), tumor progression (23, 24). However, despite several beneficial effects on the organism, cellular senescence has been reported to contribute to immune escape, drive therapeutic resistance and hamper therapeutic efficacy of cancer treatments (25–27). Thus, clarifying the senescence related biomarkers may be a promising way for the diagnosis and treatment of LUSC.

In this study, the Cancer Genome Atlas (TCGA) and Gene Expression Omnibus (GEO) databases were used to collect and evaluate RNA data and clinical information from LUSC. To create a risk model, six cellular senescence-related differentially expressed genes (DEGs) were discovered. The TCGA and GEO databases were used to divide patients into high- and low-risk groups. The high-risk group had a worse prognosis, was related with immune cell inactivation, and was less susceptible to immunotherapy, according to ROC and Kaplan-Meier analyses.

## Materials and methods

### Data processing

In this study, the TCGA database (https://portal.gdc.cancer.gov/repository) was used to retrieve the RNA-Seq dataset (n=551), mutation data (n=549), and clinical characteristics data (n=504) for LUSC. The GEO database (https://www.ncbi.nlm.nih.gov/geo/GSE73403) was used to download RNA-seq data and clinical information (n=69) for the external validation cohort.The clinical and prognostic information of immunotherapy cohort (n=348) was downloaded form IMvigor210 database (http://research-pub.gene.com/IMvigor210CoreBiologies/).

### Identification of differentially expressed genes

TCGA and GEO expression data were normalized to values of fragments per kilobase millions (FPKM), the sample’s tumor mutational load was computed as follows: the number of mutationsper million genes in each sample and the number of mutations in the sample where the gene is located were combined, a collection of senescence genes related to cell aging were downloaded from the website (https://genomics.senescence.info/cells). TCGA and GEO data were analyzed using R “limma” package, and the expression levels of overlapping genes were extracted. According to the differential expression level between normal and tumor samples of LUSC (Fold change = 1.5 and p < 0.05), 141 differentially expressed genes (DEGs) were identified.

### Immunohistochemistry analysis

To investigate the protein expression levels of the DEGs, as per the strategy described by our previous studies (28, 29), the expression of DEGs in lung cancer samples was further revealed based on the Human Protein Atlas database (https://www.proteinatlas.org/). All captured pictures were physically clarified by certified pathologists. To validate the expression of hub genes and immune microenvironment, the CDKN1A and SOX2 were further analyzed by anti-CDKN1A (CST, #2947) and anti-SOX2 (CST, #14962) in LUSC (n=4), immune cell infiltration by anti-PD-L1 (CST, #13684) and anti-FOXP3 (CST, #12653) between benign pulmonary nodule and LUSC.

### Establishment and validation senescence-related prognostic model

The gene data and clinical data of TCGA were merged using the R “limma” “survival” packages, and the genes were cycled to screen out the genes related to clinical prognosis. P < 0.05 indicated that it was a prognostic gene. The expression levels of significant genes were extracted and analyzed by univariate analysis. The HR>1 was identified as a high-risk gene, otherwise it was considered as a low-risk gene, and a total of 21 prognosis-related genes were obtained. At last, 9 genes were obtained after the intersection with 141 DEGs.

To evaluate the prognostic value of cellular senescence-related genes, LASSO regression was used to construct the clinical model using R “glmnet” “survival” packages, the model formula is output and cross-validated, and six prognostic genes were retained. The risk score was listed as following: Risk Score=∑_i_^6^Xi ×Yi (X: coefficients, Y: gene expression level). According to the risk score, high and low risk groups were identified in TCGA as the experimental group and GEO as the verification group.

### Survival analysis

To compare the survival curves of low and high risk groups, Kaplan-Meier was used. A ROC curve for the 1/3/5 years survival rate was also evaluated. Univariate and multivariate Cox regression models were used to reveal the age, stage and gender variables in the TCGA cohort. Sub-group analysis was further used to explore the risk model.

### Gene set enrichment analysis

Based on 59,428 genes and 495 samples in both the high and low risk groups, expression data and phenotypic data were collected. Using the GSEA software (http://www.gsea-msigdb.org/gsea/login.jsp), the top 100 enriched pathways in the genome were displayed. Normalized Enrichment Score > 0 was identified as high risk group, < 0 was identified as low risk group, and the low risk group was used as the control group. A total of 247 high-risk group pathway samples and 248 low-risk group pathway samples were extracted, and the top 5 standardized scores were reserved (5 in each of the high-low risk groups). R language “GGplot2” “Grid” “gridExtra” was used to evaluate the pathway activity. The chemokines, growth factors and regulators, proteases and regulators, soluble or shed receptors or ligands and interleukins were further annotated and classified.

## Tumor mutation burden

Prognosis-related cellular senescence genes and tumor mutation burden (TMB) data were processed through “ggpubr”, “survival” and “survminer”, and the intersection of the two data was taken and merged to compare the correlation between high and low risk groups, the optimal cutoff of tumor mutation burden and the difference between groups were further obtained. The survival difference and the survival curve of TMB combined with risk score were further analyzed.

### Immune cell infiltration analysis

Immune cell infiltration files (http://timer.cistrome.org) and literature summaries of immune checkpoint-related genes were downloaded the TCGA (30, 31). The immune cell infiltration, the correlation analysis, immune checkpoint gene expression, the survival analysis were analyzed useing R-packages “scales”, “ggplot2”, “ggtext”, “tidyverse”, “ggpubr”.

### Immunotherapy analysis

To validate immunotherapy value of risk score, the expression data and survival status from the IMvigor210 cohort were screen out using the R language “survival” “caret” “glmnet” “survminer” packages. The risk score according to the formula of the model were calculated, the clinical benefit status and survival difference were obtained.

### Statistical analysis

Two or more groups were compared by Wilcoxon, while differences among three or more groups were compared using Kruskal-Wallis tests and one-way analysis of variance (ANOVA). By LASSO Cox regression, receiver operating characteristic curve (ROC) analysis, and Kaplan-Meier analysis, the risk score model was constructed and evaluated. Statistical significance was determined by P<0.05 and all P values were two-sided. All data were processed using R 4.0.3 software (R Foundation for Statistical Computing, Vienna, Austria).

## Results

### Identification of prognostic senescence-related genes

**Figure 1:**
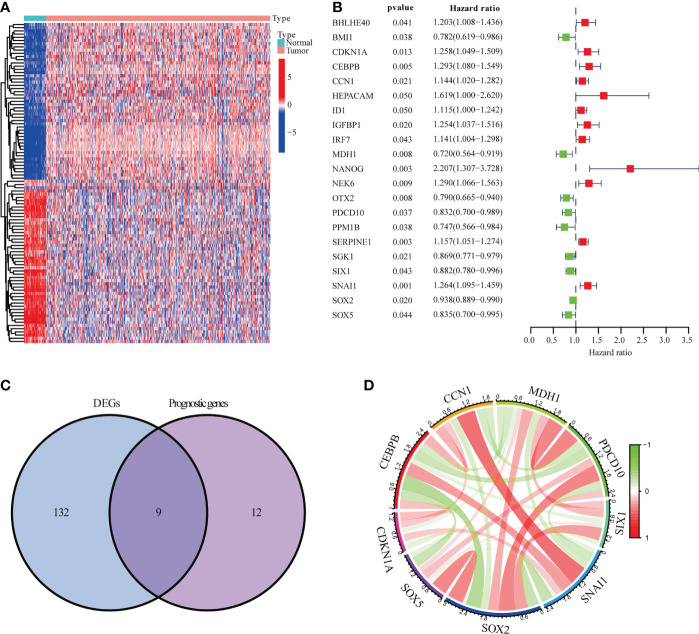
Genes linked to prognostic senescence. **(A)** The TCGA-LUSC heatmap of 141 DEGs. **(B)** The study of 21 hub genes using Univariate Cox regression. **(C)** A Venn diagram depicting DEGs linked to OS. **(D)** Correlation network of genes linked to cellular senescence.

The NSCLC data from TCGA-LUSC (n = 551) and GSE13213 (n = 69) were analyzed and shown in **Figure S1** and **Table S1**. As shown in **Figures 1A, B**, 141 DEGs between tumor (n=502) and adjacent normal tissues (n=49) and 21 prognostic genes in tumor samples were identified. Intersection analysis showed that CCN1, CDKN1A, CEBPB, MDH1, PDCD10, SIX1, SNAI1, SOX2 and SOX5 were the significant senescence-related genes (**Figure 1C**). Correlation analysis indicated that nine senescence-related genes showed a close association, such as CCN1 and SNAI1 (R=0.41), CEBPB and SOX2 (R=-0.37) (**Figure 1D** and **Table S2**).

To know the genetic alternation of the nine senescence-related genes, we firstly evaluated the copy number variations, as shown in **Figure S2A**, three copy number variations were identified in LUSC, CDKN1A, SOX5, SNAIL, CEBPB, MDH1, PACD10 and SOX2 were heterozygous amplification, while CCN1 and SIX1 were heterozygous deletion. Single nucleotide variation analysis showed the SNV frequency of the regulators was 100% among 38 LUSC samples. Variant type analysis showed that missense mutations were the main SNP type. SNV percentage analysis indicated that the mutation percentages of SOX5, SNAIL, PDCD10, SIX1, CDKN1A, SOX2 and MDH1 were 47%, 16%, 11%, 11%, 8%, 8% and 5%, respectively (**Figure S2B**). Methylation analysis indicated that SIX1, SNAIL, CCN1, CEBPB, MDH1, PDCD10 and SOX5 were significantly hypermethylated in LUSC and CDKN1A and SOX2 were significantly hypomethylated (**Figure S3**). These results indicated that the genetic alternation of senescence-related genes are mainly involve in regulating senescence in LUSC.

### Development of a senescence-related risk model

**Figure 2:**
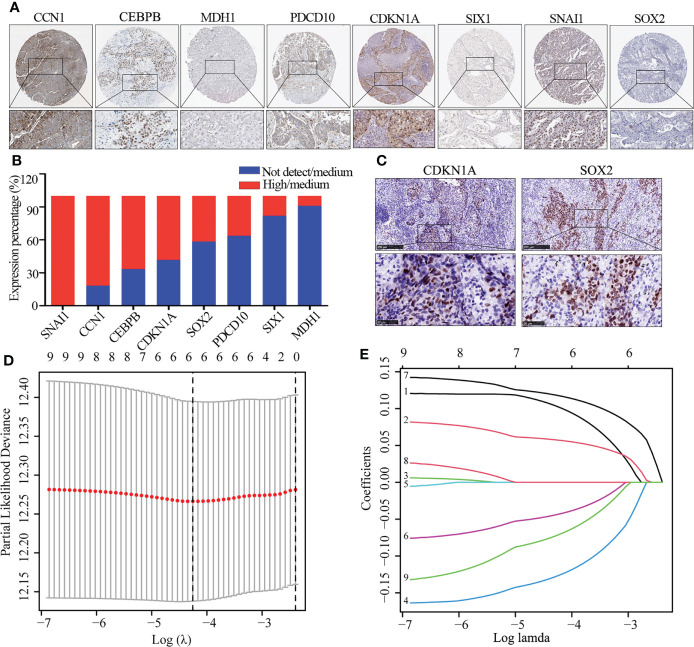
The expression of DEG and identification of hub gene. **(A, B)** Immunohistochemistry image of eight cellular senescence-related genes **(A)** and quantitative analysis **(B)**, the high and medium expression means positive expression and low and not detect means negative expression. **(C)** Immunohistochemistry image of CDKN1A and SOX2. **(D)** Regression of the six OS-related genes using LASSO. **(E)** In the LASSO regression, cross-validation is used to fine-tune the parameter selection.

We further explored the protein expression of CCN1, CDKN1A, CPBEB, MDH1, PDCD10, SIX1, SNAI1, SOX2 and SOX5. However, as there was no pathological expression of SOX5 in the human protein altas (THPA). Consistent the results of **Figure 1B**, as shown in **Figures 2A, B**, SNAI1 (n=10; 100%), CCN1 (n=11; 81.8%), CEBPB (n=12; 86.7%), CDKN1A (n=12; 58.3%) were upregulated in lung cancer, while SOX2 (n=12; 41.7%), PDCD10 (n=11; 36.4%), SIX1 (n=12; 86.7%) and MDH1 (n=11; 18.2%) were downregulated in lung cancer. Further immunohistochemistry analysis found that CDKN1A (n=4; 75%) and SOX2 (n=4; 100%) were significantly up-regulated in LUSC (**Figure 2C**). Using the LASSO analysis, Several risk (CDKN1A, CEBPB, SNAI1) and protective (MDH1, SIX1, SOX5) mRNAs were identified in patients with LUSC. Thus, a prognostic risk score formula was developed as follows: risk score = (0.103*CDKN1A exp.)+(0.057*CEBPB exp.)+(0.118* SNAI1 exp.)+(-0.127* MDH1 exp.)+(-0.044*SIX1 exp.)+ (-0.072* SOX5 exp.) (**Figures 2D, E**).

**Figure 3:**
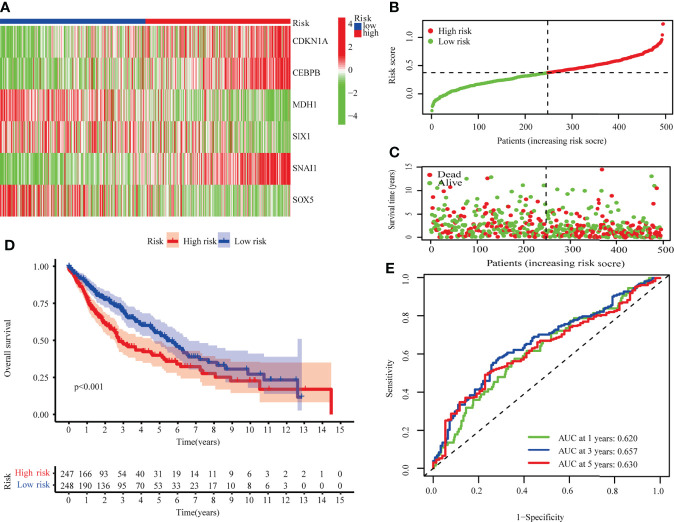
The TCGA-LUSC cohort was used to develop a risk model. **(A)** A heatmap of six hub genes involved in cellular senescence. **(B)** The TCGA cohort’s risk scores. **(C)** Survival status distribution. **(D)** Survival analysis of high and low risk groups. **(E)** The risk model’s AUC.

Risk scores were calculated for TCGA-LUSC using median scores as a cutoff value for classifying patients as high-risk (n = 247) or low-risk (n = 248). As shown in **Figures 3A–C**, the distribution of risk scores and the survival status of patients. Survival analysis showed that TCGA-LUSC in the low-risk group displayed better OS than those in the high-risk group (P < 0.001; **Figure 3D**). To determine the prognostic capacity of the formula, ROC analyses were performed, with areas under the ROC curve for 1-, 3-, and 5-year OS of 0.620, 0.65, and 0.630, respectively, implying that the risk score could be used as a biomarker of prognosis in LUSC (**Figure 3E**).

### Validation of a senescence-related risk model

**Figure 4:**
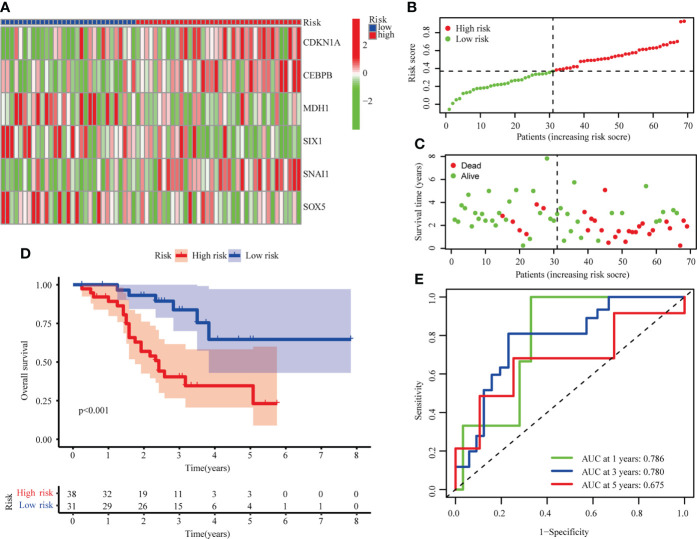
Validation of risk model in the GSE73403 cohort. **(A)** The heatmap of 6 hub cellular senescence-related genes. **(B)** The risk scores in GSE73403 cohort. **(C)** Distribution of survival status. **(D)** Survival analysis in the high risk and low risk groups. **(E)** AUC of the risk model.

LUSC from GSE73403 were analyzed in order to validate the senescence-related risk model. Patients were classified into high (n=38) and low risk (n = 31) groups, whose distributions are shown in **Figures 4A–C**. A survival analysis revealed that GSE13213 patients in the low-risk group experienced a better overall survival than their high-risk counterparts (P < 0.001; **Figure 4D**). ROC analysis showed the areas under the ROC curve for 1-, 3-, and 5-year OS were 0.786, 0.780, and 0.675 (**Figure 4E**). These result suggested the risk score could be a prognostic biomarker in patients with LUSC.

**Figure 5:**
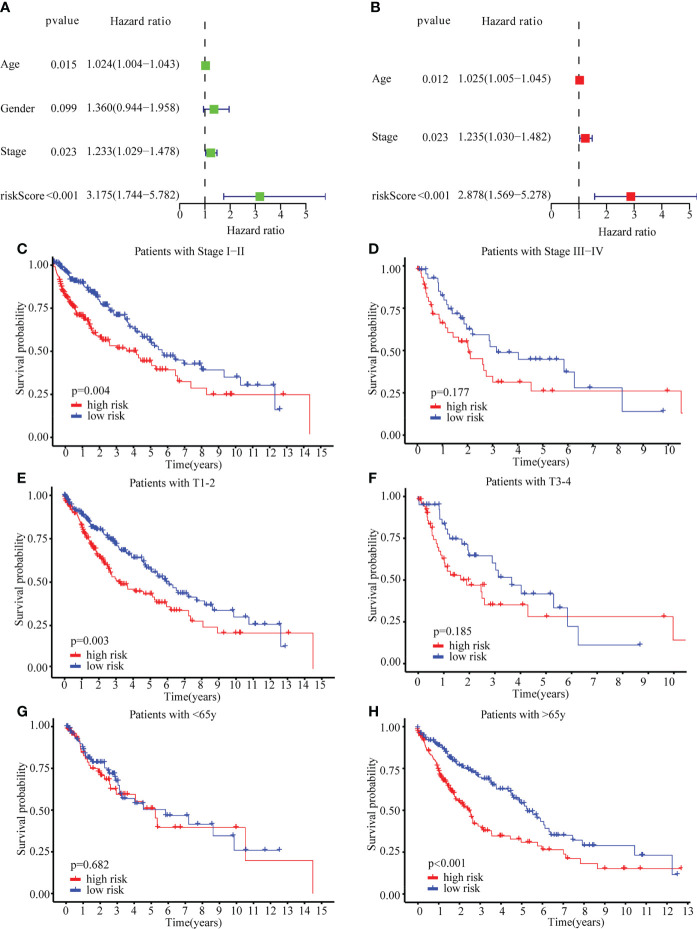
Univariate Cox regression, Multivariate Cox regression and stratified analysis. **(A)** Univariate Cox regression in TCGA cohort. **(B)** Multivariate Cox regression in TCGA cohort. **(C–H)** stratified analysis by clinical stage (I–II/III–IV) **(C, D)**, T stage (T1–2/T3–4) **(E, F)**, age (≤65/> 65) **(G, H)**.

Additionally, the risk score could be used independently to predict OS by using both univariate (HR: 3.175; 95% CI: 1.744–5.782; P < 0.001) and multivariate Cox regression (HR: 2.878; 95% CI: 1.569–5.278; P < 0.001) analysis (**Figures 5A, B**). To evaluate the clinical characteristics of the TCGA-LUSC cohort, the patients were stratified by clinical stage (I–II/III–IV), T stage (T1–2/T3–4), age (≤65/> 65). The senescence-related risk model was significantly divided into high- and low-risk subgroups based on multiple clinical characteristics, particularly I–II (P = 0.004; **Figures 5C, D**), T1–2 (P = 0.003; **Figures 5E, F**), > 65 (P < 0.001; **Figures 5G, H**). The results revealed that senescence-related risk models can be impacted by multiple clinical characteristics, leading to the heterogeneity of senescence in LUSC.

### Immune cell infiltration and immune therapy analysis

**Figure 6:**
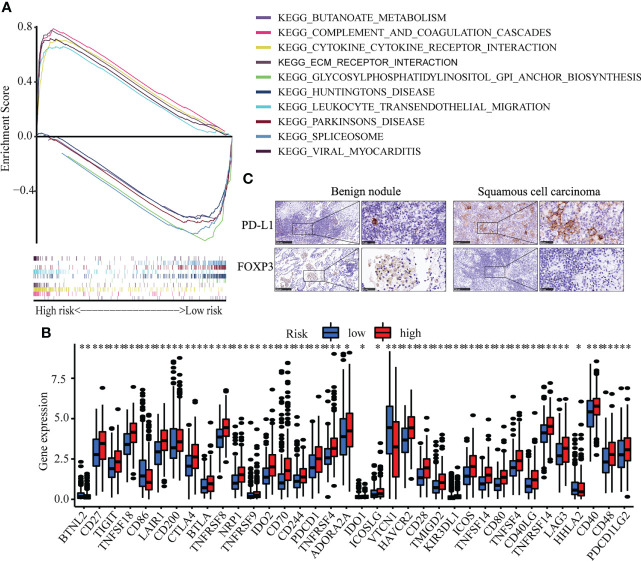
Immune cell infiltration in LUSC. **(A)** GSEA analysis between high risk and low risk groups. **(B)** The expression of immune check-point. **(C)** The Immunohistochemistry of CDKN1A and SOX2 between benign pulmonary nodule and LUSC. * means p<0.05; ** means p<0.01.

To identify the molecular function of hub senescence-related genes, GSEA enrichment analysis showed that cytokine-cytokine receptor interaction, complement and coagulation cascades, ECM-receptor interaction, viral myocarditis, leukocyte transendothelial migration were enriched in high risk groups (**Figure 6A**). Immune check-point expression analysis showed a significant correlation was observed among CD27, TIGT, TNFSF18, LAIR1, CD200, CTLA4, BTLA, TNFRSF8, NRP1, TNFRSF9, IDO2, CD70, CD244, PDCD1, TNFRSF4, ADORA2A, IDO1, ICOSLG, HAVCR2, CD28, TMIGD2, KIR3DL1, ICOS, TNFSF14, CD80, TNFSF4, CD40LG, TNFRSF14, LAG3, CD40, CD48 and PDCD1LG2 in high risk group (P<0.05; **Figure 6B**), while BTNL2, CD86, HHLA2 in low risk group (P<0.05; **Figure 6B**). These results indicated the senescence-related genes might involve in regulating tumor immune microenvironment.

**Figure 7:**
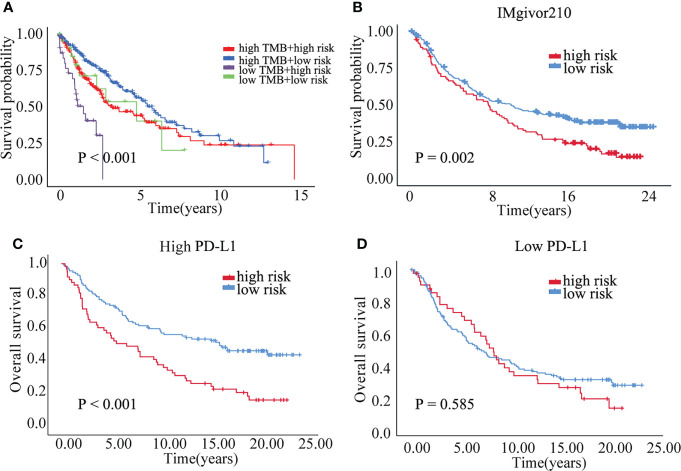
The prognostic of risk model. **(A)** The survival crosstalk between the risk score and TMB. **(B)** The prognosis between high and low risk group in IMvigor210 cohort. **(C)** The overall survival of high and low risk group in PD-L1 high expression group. **(D)** The overall survival of high and low risk group in PD-L1 low expression group.

Correlation analysis between senescence-related genes and immune cell infiltration showed that senescence-related genes were associated with distinct immune cell infiltration by CIBERSORT, EPIC, MCPCOUNTER, QUANTISEQ, TIMER and XCELL analysis (**Figures S4A–F**). The cross-talk between risk scores and immune check-points regarding patient survival was studied. The TCGA-LUSC patients could be significantly stratified by both the risk score and the immune check-point using Kaplan–Meier analysis (P<0.05; **Figure S5**). To explore the immune cell infiltration in LUSC, we further validate the expression of PD-L1 and FOXP3 between benign nodule and LUSC and found that compared with benign nodule, PD-L1 was significantly up-regulated and FOXP3 was down-regulated in LUSC (**Figure 6C**). Despite widespread use of tumor mutation burden (TMB) as a biomarker for immunotherapy, the expression of TMB did not differ significantly between high risk and low risk groups. However, the low risk/high TMB group, however, had a better survival rate than the high risk/low TMB group (P<0.05; **Figure 7A**). To explore the value of risk model, immune therapy analysis revealed that low risk had better clinical outcome than the high risk group in IMvigor210 cohort (P=0.002; **Figure 7B**). Similarly, the low risk group with PD-L1 high expression had a better overall survival than high risk group (P<0.001; **Figure 7C**). The survival of low PD-L1 expression had no survival benefit (P=0.585; **Figure 7D**). Sub-group analysis also identified that the IMvigor210 patients could be significantly stratified by both the risk score and the immune check-point using Kaplan–Meier analysis (P<0.05; **Figure S6**). These results revealed that senescence-related genes involved in immune cell infiltration and associated with the response of immune therapy.

## Discussion

Cellular senescence is a phenotype of cell cycle arrest that can be induced by different stresses (32–34). However, despite its involvement in diverse physiological processes, it has been proven that senescence inhibits tumor development in different types of cancer (35–37). In the tumor microenvironment, Senescence-associated secretory phenotypes (SASP), triggered by senescent tumor cells, cause immune cells to be recruited and activated, resulting in both antitumor and tumor-promoter actions (36–38). Herein we comprehensively performed clinical and immunological analysis of cellular senescence-related genes in LUSC. A novel risk model was developed to predict LUSC prognosis and respond to immunotherapy. The LUSC patients could be stratified by the cellular senescence-related risk model. The results of both multivariate and univariate Cox regressions revealed that the risk score may serve as a biomarker for overall survival and early diagnosis of LUSC with >65, T1-2 and stage I-II.

The risk model was composed of six senescence-related genes: CDKN1A, CEBPB, SNAI1, MDH1, SIX1 and SOX5. CDKN1A, encodes cyclin-dependent kinase inhibitor proteins p21^Cip1/Waf1^, which could activate cellular senescence (39–41). CEBPB could act as a critical determinant of cellular senescence to oncogenic Ras signaling (42). Snail, a zinc finger transcription protein, suppressed cellular senescence and promoted cancer invasion (43). Snail regulated cell survival and inhibits cellular senescence in human metastatic prostate cancer cell lines (44). Cytosolic malate dehydrogenase (MDH1) regulated senescence in human fibroblasts (45). SIX1, one member of homeobox transcriptional factors, repressed senescence by regulating cellular plasticity during tumorigenesis (46). SIX1 can also regulate cellular senescence by the regulation of p16INK4A and differentiation-related genes (47). SOX5, a member of the high-mobility group, inhibits dermal glioma formation in mice with ink4a deficiency by induction of acute cellular senescence (48).

In this study, senescence-related genes were found to control immune cell infiltration and immune treatment response in function enrichment and immunological check-point analyses. Because of its antitumorigenic and tumor-promoting properties in cancer, the SASP has been regarded as a double-edged sword. Senescence, according to recent research, can generate an immunosuppressive microenvironment that promotes cancer (49, 50). Senescent cell immunogenic switch could be identified by adaptive immune system, resulting in delayed tumor growth (11, 51, 52). In mice, macrophages recruited by CCL2 release and stimulated further by CD4+ T cells destroy Nras^G12V^-senescent premalignant hepatocytes. SASP factors produced by senescent cells aided in vascular remodelling, facilitating drug delivery and promoting the concentration of CD8+ T cells, the cytotoxicity of which may be improved by antibody-mediated PD-1 suppression. Furthermore, CDK4/6 inhibition-induced cellular senescence evoked antitumor immunity *via* regulatory T cell suppression and re-expression of endogenous retroviral elements, which provoked an interferon response (53). Our findings further indicated that tumor-specific cellular immunity was downregulated in high-risk individuals compared to low-risk patients and immunohistochemistry analysis found that FOXP3+ Treg was significantly downregulated in LUSC. Furthermore, the GSEA suggested that the immunological pathways in high-risk patients were dramatically altered. Meanwhile, the low risk group had a better survival benefit, especially with high expression of immune check-point and TMB. Thus, therapeutic interference with key factors regulating immune responses is a promising strategy to improve the clearance of premalignant senescent cells in high-risk LUSC and prevent tumour growth.

There were several drawbacks to this study as well. To begin, public data were acquired in order to create and validate a senescence-related risk model; however, prospective data from multicenter studies must be investigated further. Second, the immune cell infiltration mediated by senescence-related genes in TME need be further explored *in vivo*. Third, the risk model was constructed based on senescence-related genes, the diagnostic performance need be further improved combined with clinical index. Last, function and mechanism of senescence-related genes were theoretical, the concrete mechanism need to be further explored.

## Conclusion

In conclusion, we systematically analyzed the clinical and immunological characteristics of cellular senescence-related genes in LUSC. We developed and validated a novel senescence-related risk model that can serve as a biomarker for prognosis and clinical immune therapy. Targeting senescence-related genes may be an alternative way to improve clinical therapy for LUSC.

## Data availability statement

The original contributions presented in the study are included in the article/**Supplementary Material**. Further inquiries can be directed to the corresponding authors.

## Author contributions

XH, GL, JZ and JW conceptualized the study, created the figures, and wrote the text. Data was collected, analyzed, and the results were interpreted by LG, ZD, LW, and JZ. The study was designed by XH and JZ. All authors contributed to the article and approved the submitted version.

## Funding

This study was supported by grants from the National Natural Science Foundation of China (82003212) and the Discipline Construction Project of Guangzhou Medical University during the 14th Five-Year Plan (06-410-2107181).

## Conflict of interest

The authors declare that the research was conducted in the absence of any commercial or financial relationships that could be construed as a potential conflict of interest.

## Publisher’s note

All claims expressed in this article are solely those of the authors and do not necessarily represent those of their affiliated organizations, or those of the publisher, the editors and the reviewers. Any product that may be evaluated in this article, or claim that may be made by its manufacturer, is not guaranteed or endorsed by the publisher.

## Supplementary material

Supplementary Figure 1 The work flow of this study.

Supplementary Figure 2 Correlation analysis between senescence-related genes and immune cell infiltration. **(A–F)** Correlation analysis by CIBERSORT **(A)**, EPIC **(B)**, MCPCOUNTER **(C)**, QUANTISEQ **(D)**, TIMER **(E)** and XCELL **(F)** analysis.

Supplementary Figure 3 The copy number variations **(A)** and single nucleotide variation **(B)** of senescence-related genes.

Supplementary Figure 4 The methylation status of senescence-related genes.

Supplementary Figure 5 Survival analysis stratified by both the risk score and the immune check-point in the TCGA-LUSC cohort.

Supplementary Figure 6 Survival analysis stratified by both the risk score and the immune check-point in the IMvigor210 cohort.

Supplementary Table 1 The clinicopathological features of LUSC.

Supplementary Table 2 Correlation among 9 cellular senescence-related genes.
