# Supplementary figures and images for: Novel cellular senescence-related risk model identified as the prognostic biomarkers for lung squamous cell carcinoma
Source: Front Oncol. 2022 Nov 17;12:997702. doi: 10.3389/fonc.2022.997702 (PMC9712184; doi:10.3389/fonc.2022.997702)

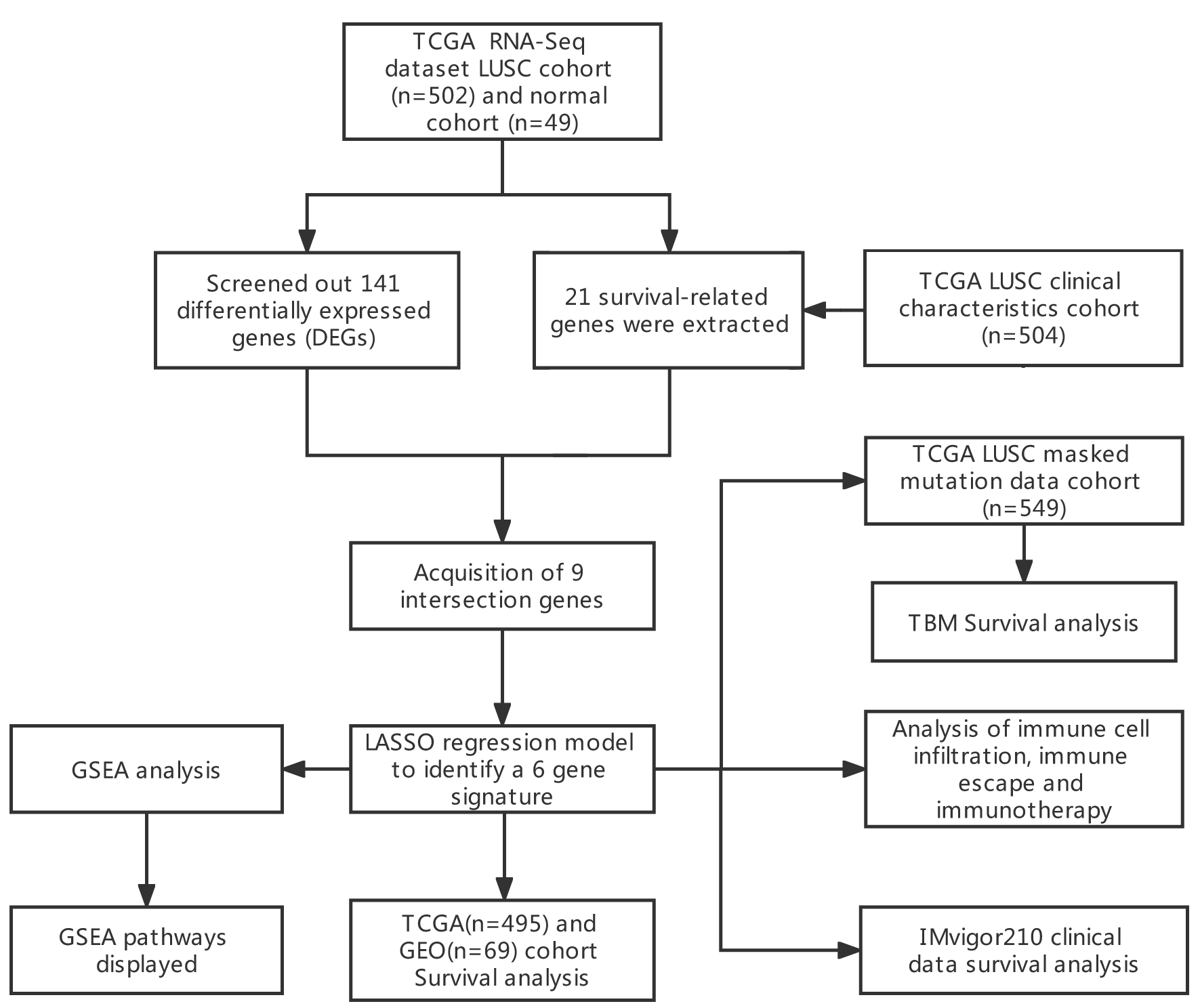

Supplement: Supplementary Figure 1 — The work flow of this study. [file Image_1.tif]

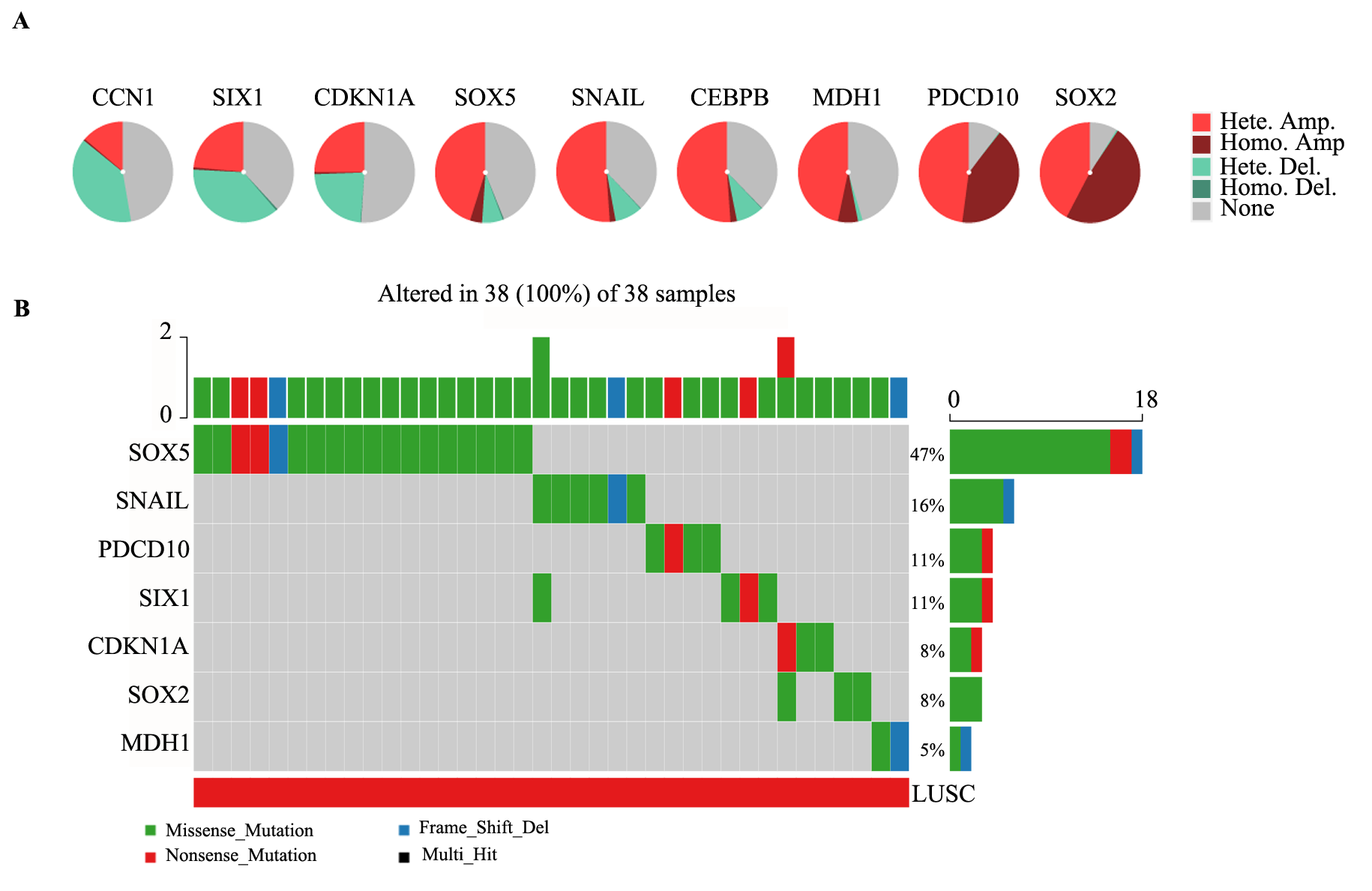

Supplement: Supplementary Figure 2 — Correlation analysis between senescence-related genes and immune cell infiltration. (A–F) Correlation analysis by CIBERSORT (A), EPIC (B), MCPCOUNTER (C), QUANTISEQ (D), TIMER (E) and XCELL (F) analysis. [file Image_2.tif]

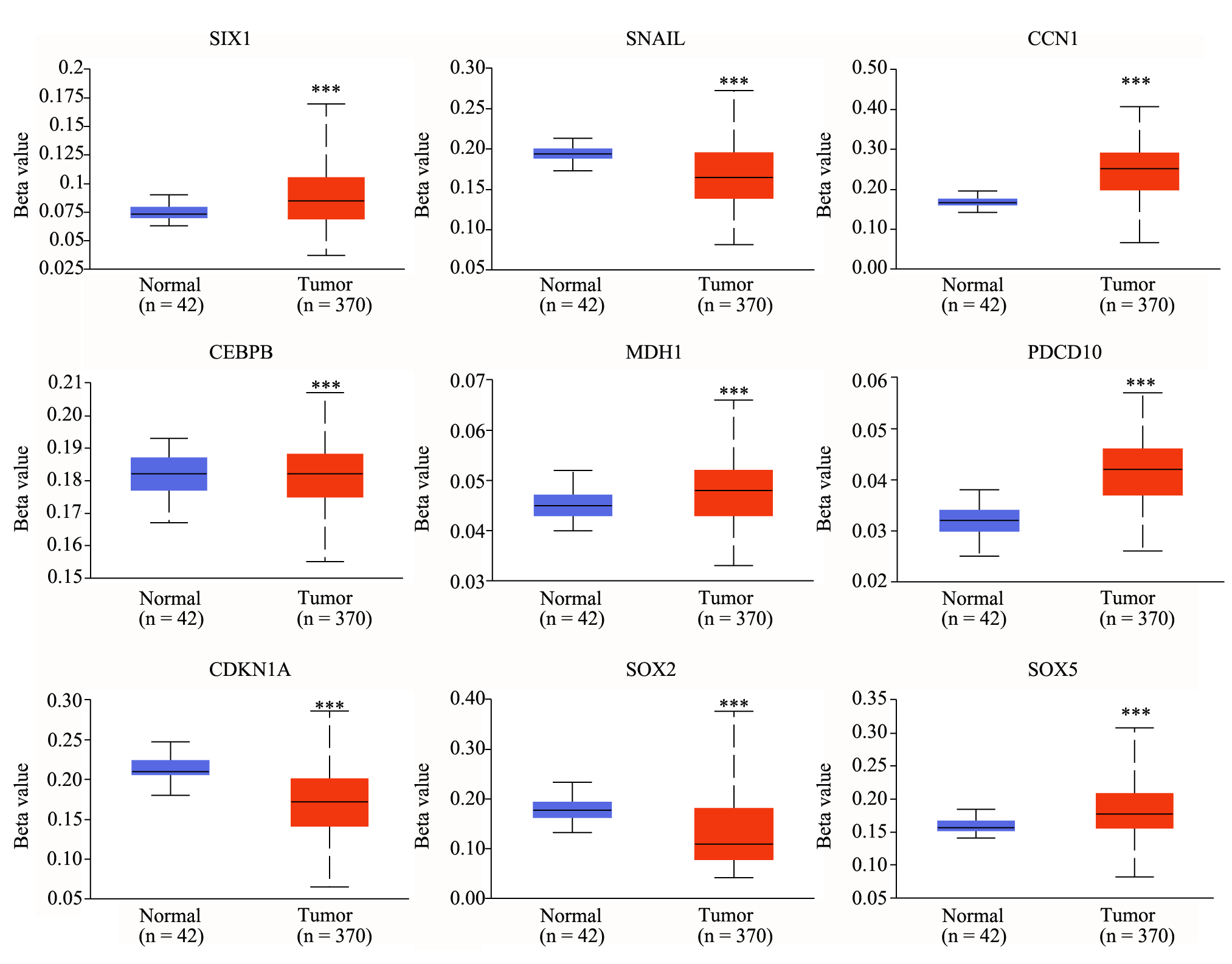

Supplement: Supplementary Figure 3 — The copy number variations (A) and single nucleotide variation (B) of senescence-related genes. [file Image_3.tif]

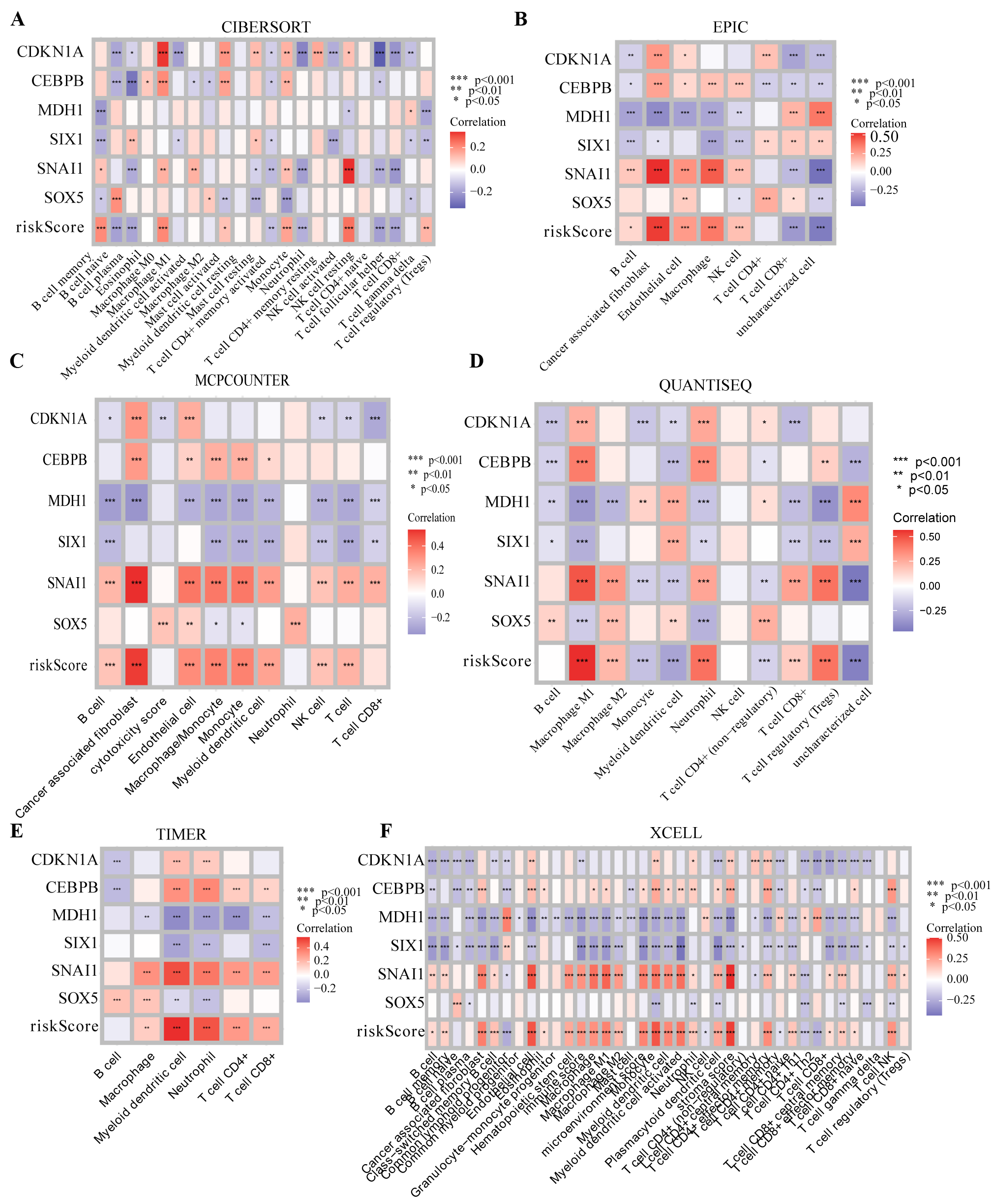

Supplement: Supplementary Figure 4 — The methylation status of senescence-related genes. [file Image_4.tif]

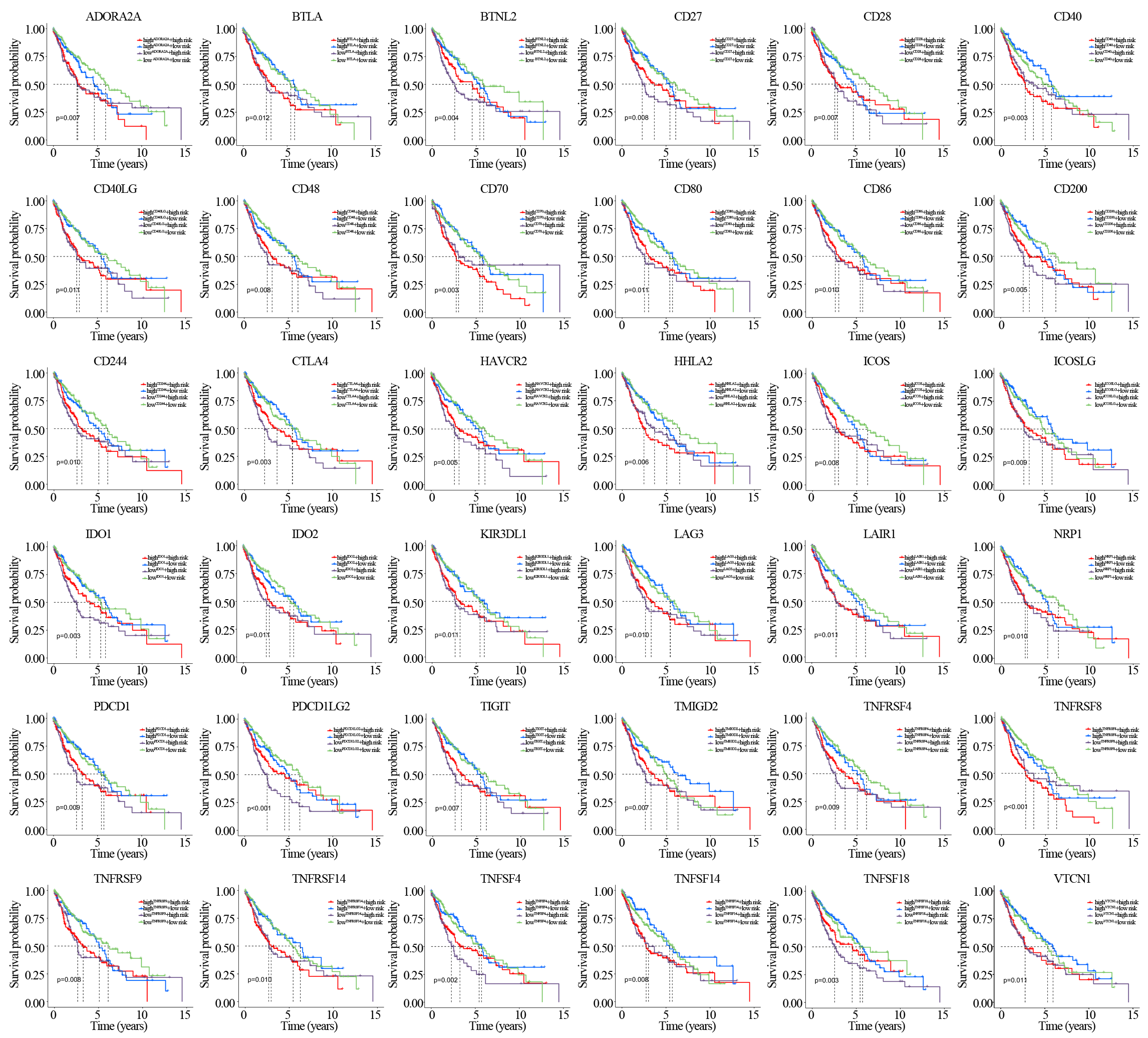

Supplement: Supplementary Figure 5 — Survival analysis stratified by both the risk score and the immune check-point in the TCGA-LUSC cohort. [file Image_5.tif]

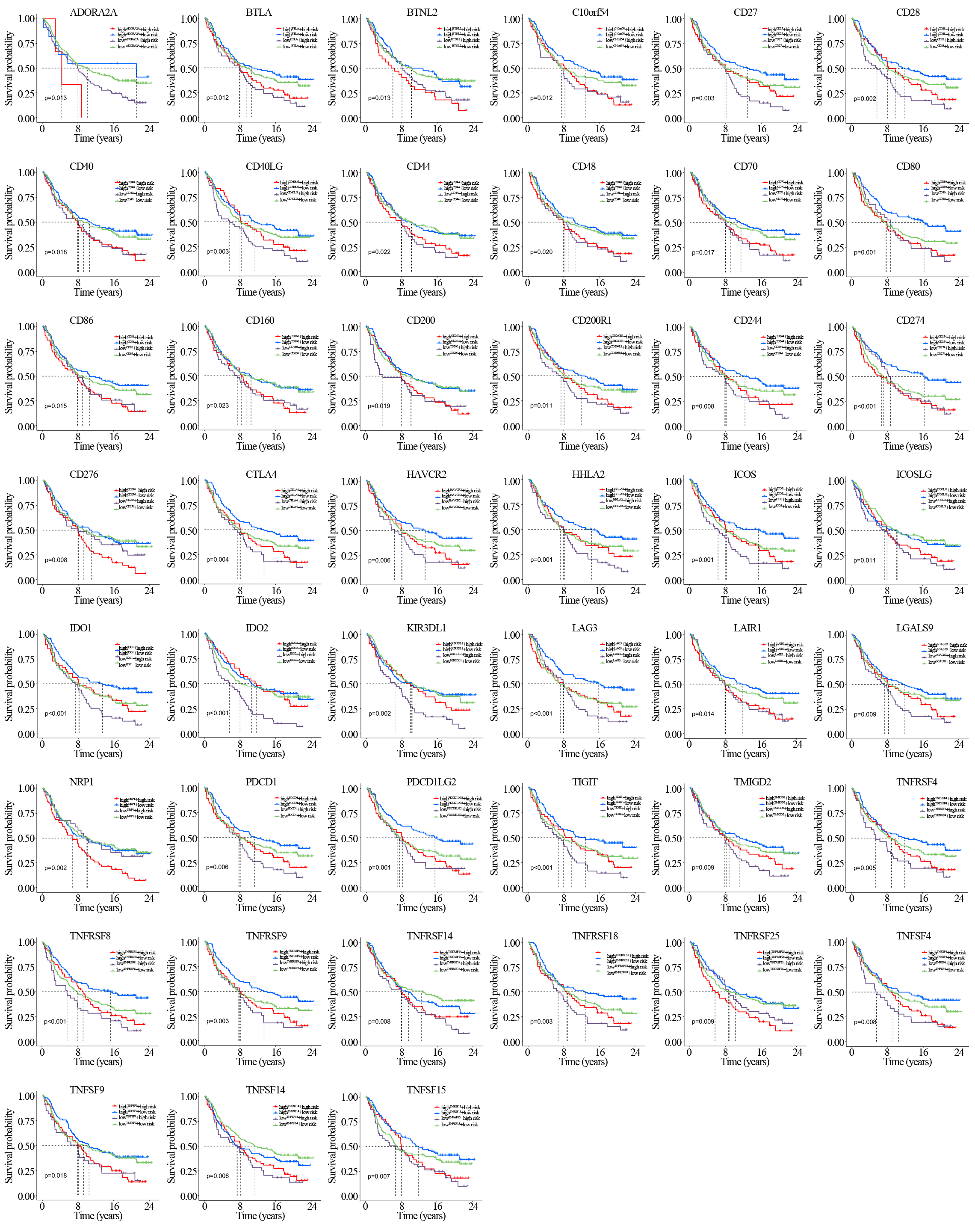

Supplement: Supplementary Figure 6 — Survival analysis stratified by both the risk score and the immune check-point in the IMvigor210 cohort. [file Image_6.tif]
