# Supplementary material for: Novel cellular senescence-related risk model identified as the prognostic biomarkers for lung squamous cell carcinoma
Source: Front Oncol. 2022 Nov 17;12:997702. doi: 10.3389/fonc.2022.997702 (PMC9712184; doi:10.3389/fonc.2022.997702)
Supplement: Supplementary Table 1 — The clinicopathological features of LUSC. [file Table_1.docx]

| **Table S1** The clinicopathological features of LUSC | | |
| --- | --- | --- |
| **Variable** | **TCGA-LUSC cohort** | **GSE73403 cohort** |
|  | **(n=504)** | **(n=69)** |
| Age |  |  |
| ≤65 | 190 | 47 |
| ＞65 | 305 | 22 |
| Age（unknow） | 9 | 0 |
| Gender |  |  |
| Female | 131 | 65 |
| Male | 373 | 4 |
| Stage |  |  |
| Stage I-II | 408 | 46 |
| Stage III-IV | 92 | 23 |
| Stage unknow | 4 | 0 |
| T |  |  |
| T1-2 | 409 | 46 |
| T3-4 | 95 | 23 |
| N |  |  |
| N0/NX | 326 | 35 |
| N1 | 133 | 17 |
| N2 | 40 | 17 |
| N3 | 5 | 0 |
| M |  |  |
| M0/MX | 497 | 69 |
| M1 | 7 | 0 |
